# FERN: is it possible to conduct a randomised controlled trial of intervention or expectant management for early-onset selective fetal growth restriction in monochorionic twin pregnancy – protocol for a prospective multicentre mixed-methods feasibility study

**DOI:** 10.1136/bmjopen-2023-080021

**Published:** 2024-08-17

**Authors:** Asma Khalil, Smriti Prasad, Kerry Woolfall, Tracy Karen Mitchell, Jamie J Kirkham, Odai Yaghi, Tracey Ricketts, George Attilakos, Carolyn Bailie, Christine Cornforth, Mark Denbow, Louise Hardman, Jane Harrold, Rajeswari Parasuraman, Shauna Leven, Joel Marsden, Jessica Mendoza, Tommy Mousa, Surabhi Nanda, Baskaran Thilaganathan, Mark Turner, Michelle Watson, Karen Wilding, Mariana Popa, Zarko Alfirevic, Dilly Anumba, Richard Edmund Ashcroft, Ahmet Baschat, Fabrício da Silva Costa, Jan Deprest, Natasha Fenwick, Monique C Haak, Andy Healey, Kurt Hecher, Lawrence Impey, Richard J Jackson, Edward D Johnstone, Liesbeth Lewi, Enrico Lopriore, Aris T Papageorghiou, Dharmintra Pasupathy, Jane Sandall, Andrew Sharp, Shakila Thangaratinam, Brigitte Vollmer, Yoav Yinon

**Affiliations:** 1 Fetal Medicine Unit, St George's University Hospital, London, UK; 2 Fetal Medicine Unit, Liverpool Women’s Hospital, University of Liverpool, Liverpool, UK; 3 Vascular Biology Research Centre, Molecular and Clinical Sciences Research Institute, St George's University of London, London, UK; 4 Department of Public Health, Policy and Systems, Institute of Population Health, University of Liverpool, Liverpool, UK; 5 Centre for Biostatistics, The University of Manchester, Manchester Academic Health Science Centre, Manchester, UK; 6 Department of Women’s and Children’s Health, Faculty of Health & Life Sciences, Harris Wellbeing of Women Research Centre, University of Liverpool, Liverpool, UK; 7 Women's Health Division, University College London Hospitals NHS Foundation Trust, Institute for Women's Health, University College London, London, UK; 8 Royal Jubilee Maternity Hospital, Belfast, UK; 9 Fetal Medicine Unit, St Michael's Hospital, University Hospitals Bristol NHS Foundation Trust, Bristol, UK; 10 Liverpool Women’s NHS Foundation Trust, Liverpool, UK; 11 Wessex Fetal Maternal Medicine unit, University Southampton NHS Foundation Trust, Princess Anne Hospital, Southampton, UK; 12 Twins Trust, The Manor House, Aldershot, UK; 13 PPIE, FERN project, Liverpool, UK; 14 University of Leicester, Leicester, UK; 15 Guy's and St Thomas's Hospital, London, UK; 16 Clinical Directorate, Faulty of Health and Life Sciences, University of Liverpool, Liverpool, UK; 17 Academic Unit of Reproductive and Developmental Medicine, Department of Human Metabolism, University of Sheffield, Sheffield, UK; 18 School of Law, City University of London, London, UK; 19 Johns Hopkins Center for Fetal Therapy Department of Gynecology & Obstetrics, Johns Hopkins University, Baltimore, Maryland, USA; 20 Maternal Fetal Medicine Unit, Gold Coast University Hospital, Gold Coast, Queensland, Australia; 21 Fetal Medicine Unit, Dept. Obstetrics and Gynecology, University Hospitals Leuven, Leuven, Belgium; 22 Dept of Development and Regeneration, Biomedical Sciences, KU Leuven, Leuven, Belgium; 23 Obstetrics and Gynaecology, Leiden University Medical Center, Leiden, Netherlands; 24 King's Health Economics, Health Service, and Population Research Department, King's College London, London, UK; 25 Department of Obstetrics and Fetal Medicine, University Medical Center Hamburg-Eppendorf, Hamburg, Germany; 26 Department of Fetal Medicine, John Radcliffe Hospital, Oxford University Hospitals NHS Foundation Trust, Oxford, UK; 27 Department of Statistics, Liverpool Clinical Trials Unit, University of Liverpool, Liverpool, UK; 28 Maternal and Fetal Health Research Centre, Division of Developmental Biology and Medicine, School of Medical Sciences, Faculty of Medicine Biology and Health, University of Manchester, Manchester, UK; 29 Department of Paediatrics, Division of Neonatology, Leiden University Medical Center, Leiden, Netherlands; 30 Nuffield Department of Women’s & Reproductive Health, University of Oxford, Oxford, UK; 31 Reproduction and Perinatal Centre, Faculty of Medicine and Health, The University of Sydney, Sydney, New South Wales, Australia; 32 Division of Women's Health, Women's Health Academic Centre, King's College, London, St. Thomas' Hospital, London, UK; 33 WHO Collaborating Centre for Global Women’s Health, Institute of Metabolism and Systems Research, University of Birmingham, Birmingham, UK; 34 NIHR Biomedical Research Centre, University Hospitals Birmingham, Birmingham, UK; 35 Birmingham Women’s and Children’s NHS Foundation Trust, Birmingham, UK; 36 Clinical Neurosciences, Faculty of Medicine, University of Southampton, Southampton, UK; 37 Department of Obstetrics and Gynecology, Chaim Sheba Medical Center, Tel-Hashomer, Ramat-Gan 52621, Israel

**Keywords:** SURGERY, Mortality, Clinical Trial, Fetal medicine, OBSTETRICS

## Abstract

**Abstract:**

**Introduction:**

Selective fetal growth restriction (sFGR) in monochorionic twin pregnancy, defined as an estimated fetal weight (EFW) of one twin <10th centile and EFW discordance ≥25%, is associated with stillbirth and neurodisability for both twins. The condition poses unique management difficulties: on the one hand, continuation of the pregnancy carries a risk of death of the smaller twin, with a high risk of co-twin demise (40%) or co-twin neurological sequelae (30%). On the other, early delivery to prevent the death of the smaller twin may expose the larger twin to prematurity, with the associated risks of long-term physical, emotional and financial costs from neurodisability, such as cerebral palsy.

When there is severe and early sFGR, before viability, delivery is not an option. In this scenario, there are currently three main management options: (1) expectant management, (2) selective termination of the smaller twin and (3) placental laser photocoagulation of interconnecting vessels. These management options have never been investigated in a randomised controlled trial (RCT). The best management option is unknown, and there are many challenges for a potential RCT. These include the rarity of the condition resulting in a small number of eligible pregnancies, uncertainty about whether pregnant women will agree to participate in such a trial and whether they will agree to be randomised to expectant management or active fetal intervention, and the challenges of robust and long-term outcome measures. Therefore, the main objective of the FERN study is to assess the feasibility of conducting an RCT of active intervention vs expectant management in monochorionic twin pregnancies with early-onset (prior to 24 weeks) sFGR.

**Methods and analysis:**

The FERN study is a prospective mixed-methods feasibility study. The primary objective is to recommend whether an RCT of intervention vs expectant management of sFGR in monochorionic twin pregnancy is feasible by exploring women’s preference, clinician’s preference, current practice and equipoise and numbers of cases. To achieve this, we propose three distinct work packages (WPs). WP1: A Prospective UK Multicentre Study, WP2A: a Qualitative Study Exploring Parents’ and Clinicians’ Views and WP3: a Consensus Development to Determine Feasibility of a Trial. Eligible pregnancies will be recruited to WP1 and WP2, which will run concurrently. The results of these two WPs will be used in WP3 to develop consensus on a future definitive study. The duration of the study will be 53 months, composed of 10 months of setup, 39 months of recruitment, 42 months of data collection, and 5 months of data analysis, report writing and recommendations. The pragmatic sample size for WP1 is 100 monochorionic twin pregnancies with sFGR. For WP2, interviews will be conducted until data saturation and sample variance are achieved, that is, when no new major themes are being discovered. Based on previous similar pilot studies, this is anticipated to be approximately 15–25 interviews in both the parent and clinician groups. Engagement of at least 50 UK clinicians is planned for WP3.

**Ethics and dissemination:**

This study has received ethical approval from the Health Research Authority (HRA) South West—Cornwall and Plymouth Ethics Committee (REC reference 20/SW/0156, IRAS ID 286337). All participating sites will undergo site-specific approvals for assessment of capacity and capability by the HRA. The results of this study will be published in peer-reviewed journals and presented at national and international conferences. The results from the FERN project will be used to inform future studies.

**Trial registration number:**

This study is included in the ISRCTN Registry (ISRCTN16879394) and the NIHR Central Portfolio Management System (CPMS), CRN: Reproductive Health and Childbirth Specialty (UKCRN reference 47201).

**STRENGTHS AND LIMITATIONS OF THIS STUDY**

- The research question—acceptability and feasibility of this proposed trial has not been investigated previously.
- Uses a mixed-methods design incorporating prospective multicentre, qualitative and consensus-building approaches to enhance methodological robustness and rigorous multifaceted investigation.
- Includes a prospective UK multicentre study (work package 1) that systematically gathers data across various centres to ensure diverse input and data richness.
- Employs qualitative interviews to deepen understanding of participant and clinician perspectives, enriching the data’s contextual validity.
- Given the rarity of the condition under study, the study may face challenges in achieving sufficient power to fully explore certain perinatal outcomes.

## Introduction

 Twin pregnancy is associated with an increased risk of adverse fetal and neonatal outcomes, such as stillbirth and neonatal death compared with singletons.^1^ Twin pregnancies are also at fourfold increased risk of neurological sequelae such as cerebral palsy compared with singletons.^2^ Monochorionic (MC) twin pregnancies, where both fetuses originate from the same conception and share one placenta, represent 25%–30% of all twin pregnancies.^3^ MC twin pregnancies pose unique difficulties for management because of complications from a shared placenta and communication between the fetal circulations. These can lead to the two common pathologies seen in MC twin pregnancies: twin-to-twin transfusion syndrome (TTTS) and selective fetal growth restriction (sFGR). sFGR, when one fetus grows normally while the other is growth restricted, affects approximately 10%–15% of MC twin pregnancies.

**Figure 1:**
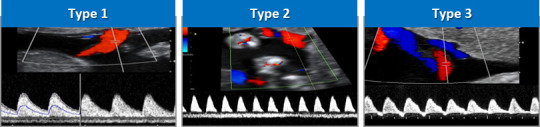
Classification of selective fetal growth restriction according to umbilical artery flow patterns.

Definitions of sFGR in MC twin pregnancy vary, but current UK guidelines define sFGR as an intertwin estimated fetal weight (EFW) discordance of >20% after 20 weeks.^3 4^ The ISUOG (International Society of Ultrasound in Obstetrics and Gynecology) guideline supports using the smaller twin being <10th centile and an intertwin discordance of ≥25%0.^5^ Early-onset sFGR, occurring prior to 24 weeks’ gestation, is less common but is associated with greater risk to the fetus and poses substantial management difficulties due to the distance from viability.^6^ sFGR in MC twin pregnancy is conventionally classified into three subtypes based on the umbilical artery (UA) Doppler (figure 1) according to Gratacós *et al*: type I (positive end-diastolic flow (EDF) in the UA) has the best outcome, type II (persistent absent/reversed EDF) the worst prognosis and type III (intermittently absent/reversed EDF) has an unpredictable course.^7^

There are three main management options for sFGR:

(1) Expectant management: close monitoring until the decision for timed birth is made. This carries a risk of intrauterine demise (IUD) of the smaller twin, which may result in co-twin demise (40%) or neurological sequelae (30%), thought to be secondary to hypotension in the larger twin due to intertwin transfusion via placental vascular connections.^3^ (2) Selective termination by bipolar cord coagulation (BCC) or radiofrequency ablation (RFA). This may protect the larger baby from harm if the smaller twin were to subsequently die. However, termination may not be acceptable to some parents. (3) Placental laser photocoagulation of connecting vessels to protect the larger twin in the event of death of the smaller twin. This is often technically challenging and may worsen the outcomes of the smaller twin.^8^,^11^ Active intervention options include selective termination through either BCC or RFA, as well as placental laser photocoagulation of connecting vessels.

We conducted a preliminary survey of 29 UK fetal medicine clinicians to establish their current practice for the management of sFGR in MC twin pregnancies.^12^ Our survey revealed significant variation in diagnostic criteria, management and surveillance protocols for the three different types of early-onset sFGR in MCDA (Monochorionic diamniotic) twin.^12^ Great uncertainty also remains over what interventions, if any, women would be willing to accept at different gestations. The current paucity of qualitative data and patient experience information may hamper a subsequent clinical trial.^12^ The loss of a fetus is associated with long-term psychological effects for the parents.^13^ Furthermore, the lifelong cost of prematurity-associated neurodisability, such as cerebral palsy, may be substantial.^14^,^18^

Therefore, there is a lack of consensus on the optimal management (expectant or intervention) of sFGR in MC twin pregnancies and the most appropriate timing of delivery. Each management option carries risks and benefits in terms of the key outcomes of stillbirth and cerebral palsy; what is currently unknown is the relative risk–benefit ratio for each option.

No randomised controlled trials (RCT) or Cochrane reviews have investigated sFGR. Until recently, published systematic reviews^9 11^ assessed mortality and morbidity, especially cerebral injury,^18^ but did not compare outcomes based on management. We have published a meta-analysis of the perinatal mortality and morbidity in sFGR according to management^8^ which demonstrated significant bias in the published literature. Studies were retrospective and non-randomised, with significant heterogeneity in included populations, management and outcomes.^8^ This meta-analysis was, therefore, unable to explore the association between gestational age at delivery and neonatal outcomes. This is fundamental as gestational age at delivery is the main determinant of perinatal outcome in twins.^17 18^ Primary research has not identified a significant difference between RFA and BCC for selective fetal reduction for sFGR in MC twin pregnancy.^19 20^ In summary, to quote the Royal College of Obstetricians and Gynaecologists,^3^ ‘Due to a lack of available high-quality evidence, there is no clear guidance on how to manage sFGR in such pregnancies.’

The primary objective is to recommend whether an RCT of intervention versus expectant management of sFGR in MC twin pregnancy is feasible by exploring women’s preference, clinician’s preference, current practice and equipoise and numbers of cases.

## Methods and analysis

### Study design

**Figure 2:**
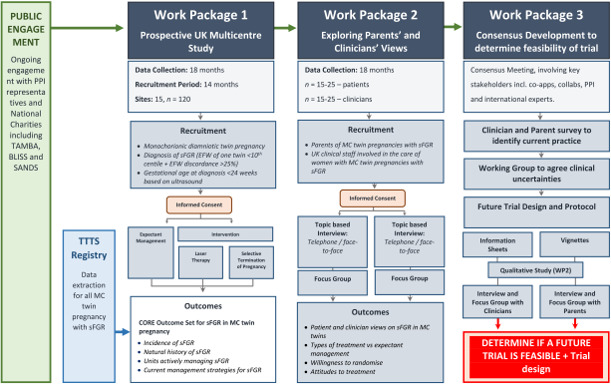
FERN study flow chart.

FERN is a mixed-methods feasibility study with three distinct work packages (WPs) to determine the overall research objectives (figure 2).

### WP 1: a prospective UK multicentre study

We will conduct a prospective UK multicentre study to determine the incidence, natural history and outcomes of MCDA twin pregnancies complicated by sFGR according to whether they had expectant management or fetal intervention.

The data will provide the following key outcomes:

1. Proportion of women who opt for expectant management, active fetal intervention or termination of the entire pregnancy. We will have reasonable precision of these estimates from current routine practice, which is critical for any future research including an RCT.
2. We will learn about management of these pregnancies, that is, frequency of monitoring, timing of delivery, thresholds for intervention.
3. Incidence of adverse outcomes. However, some subgroups may be small so the precision of some estimates might be inaccurate, with wide 95% CIs. We will use the data from the UK twin registry to ascertain the accuracy of these estimates.

#### Eligibility criteria

Women with an MCDA twin pregnancy and a diagnosis of sFGR (EFW of one twin <10th centile+EFW discordance ≥25%) and gestational age at diagnosis between 16^+0^ and 23^+6^ weeks based on ultrasound and who can provide informed consent will be considered eligible for WP1. The exclusion criteria include higher order multiple pregnancies, maternal age under 18 years, other MC complications (such as TTTS or twin anaemia polycythaemia sequence before enrolment), other rare complicated MC twin pregnancies at enrolment (such as twin reversed arterial perfusion syndrome, known karyotype abnormality, known major fetal structural abnormality defined as a lethal, incurable or curable severe abnormality with a high risk of residual disability), an indication for immediate delivery, preterm prelabour rupture of membranes before enrolment. In addition, women who lack the capacity to give informed consent or suffer from any medical or psychiatric condition which compromises their ability to participate will also be excluded.

#### Recruitment and sampling

We will approach all women with sFGR as defined by the eligibility criteria. Further to two no-cost extensions granted by the NIHR due to delayed setup and recruitment and rarity of the condition under study, there will be a 42-month data collection period (39-month recruitment) from 22 UK sites, (compared with the originally proposed 18-month data collection period). The planned start and end dates for the study are 1 July 2021 and 30 November 2025, respectively. Our recent survey suggests that we should be able to recruit two women per month per centre; if we assume a conservative 40% recruitment rate, our pragmatic estimate is that we will recruit over 100 women in the study period. All participants will be recruited from their local fetal medicine or antenatal clinic. They will be contacted in person by a member of the clinical research team (principal investigator (PI)/research midwife/delegated authority) and invited to take part in the study. Potential participants will be given written and verbal information on the FERN study and provided with the opportunity to ask questions and take any additional time required to consider taking part in the study. Following this, participants will be asked to sign the study-specific informed consent form in the presence of the site researcher. Once eligibility has been confirmed by the PI at the site, and informed consent has been obtained, the participant will be registered and then entered into the study using a bespoke electronic data capture system that will generate a unique participant identification number. The data will be collected on a range of data items described on the FERN REDCap database. The follow-up will continue until of discharge of baby/babies from the hospital/neonatal unit. This database is designed and maintained by the University of Liverpool, IT Services in collaboration with the chief investigator and research manager. The eCRF (electronic case record form) is the primary data collection instrument for the study. All data requested on the eCRF must be recorded and all missing data explained. WP1 is a prospective observational study, therefore, all data will be collected prospectively by trained members of the research team at each study site.

### Sample size calculation

No formal power calculation or primary outcome data will be determined for any WP in this study as this is a feasibility study and does not involve any formal hypothesis testing. We will assess the feasibility of recruiting women with an MC twin pregnancy complicated by sFGR to collect detailed information about pregnancy management and outcomes. The success of this WP will be determined by the acceptability of the study by women and clinicians. This will be determined as the ability to recruit and retain participants. We would expect to recruit in the region of 100 participants across the UK over a 39-month period, but we will continue to recruit until the recruitment period is complete. This is a pragmatic number which is based on the typical number of sFGR patients seen per annum in large consultant-led NHS units within the UK.

### Patient and public involvement

No patient and public involvement was sought during the set up or design of this feasibility study.

### Outcome measures

This study will assess the feasibility of collecting outcome data from women with an MCDA twin pregnancy complicated by sFGR.

### WP 2: exploring patients’ and clinicians’ views

This will involve qualitative research, including interviews and focus groups (if there is divergence of opinion about trial acceptability) with women and their partners (if applicable), as well as with clinicians involved in the management of MC twin pregnancies. Qualitative methods will explore parents’ and clinicians’ perspectives on:

1. Future trial design including views on active intervention and expectant management, randomisation, outcomes, and approach to recruitment and consent, including decision-making and length and content of trial information materials.
2. Factors influencing parent and clinician decision-making when potential outcomes include death or serious disability of one or both twins.
3. Acceptability of a future trial, including potential barriers to recruitment, consent decisions, trial procedures, equipoise; inclusion/exclusion criteria and training needs.

#### Eligibility criteria

Mothers and partners (if applicable) of MCDA twin pregnancies complicated by sFGR in the previous 3 years; and clinical staff involved in the management of MC twin pregnancies.

#### Recruitment and sampling

Women and their partners (if applicable) will be recruited through two routes to maximise the potential participation within the recruitment period and ensure that women from all management groups are represented. First, all women and partners (if applicable) approached to take part in WP1 will be invited to consent to be contacted by a member of the WP2 research team to discuss taking part in this aspect of the study, regardless of ultimate participation in WP1. Second, the qualitative research team will contact stakeholders (eg, charity leads/chief executive officers) of twin support groups (eg, Ttwins Trust—formerly Twins and Multiple Births Association) inviting them to post a FERN study media advert on the support group’s website and/or social media pages (eg, Facebook and Twitter) or member email lists. If divergence in opinions on trial intervention and acceptable outcomes is observed in the early analysis of interviews, we will use social media (route 2) to recruit parents to a focus group (~6–10 parents) in the Northwest of England (pragmatic choice as location of the qualitative study team) with the aim of reaching a consensus about an acceptable trial design. A focus group topic guide will be developed based on interview findings.

For clinician recruitment, a comprehensive database of clinicians, nationally and internationally, who lead the management of sFGR will be developed by the CI, coapplicant team and collaborators. This database will be used to identify which clinicians will be invited to take part in this aspect of the study. Second, an FERN practitioner online recruitment advert will be posted on relevant social media pages. As with the women and partner interviews, if divergence in opinions is observed in the early analysis of interviews, clinicians will be recruited to a focus group (~8–10 site healthcare professionals) with the aim of reaching consensus about an acceptable trial design. A focus group topic guide will be developed based on interview findings.

#### Sample size calculation

To ensure sample variance, we will include parents with experience of intervention and expectant management of sFGR, bereaved and non-bereaved parents, and clinicians in favour of both fetal intervention and expectant management. Interviews will be conducted till we achieve information power,^21^ which is the point at which data addresses the study aims; sample variance (eg, parents offered expectant management or intervention, bereaved and non-bereaved parents, and clinicians in favour of intervention or expectant management) and, use of theory (eg, bioethical principles) and quality of dialogue.

#### Primary outcome measures

Qualitative element of the study will explore parents’ and clinicians’ perspectives and present a summary of findings and recommendations about trial design/feasibility to feed into WP3.

### WP 3: consensus development to determine feasibility of a trial

We will use the information provided in WP1 and WP2 to develop a consensus on a future definitive study. The effect of a design of a future RCT, if deemed acceptable and feasible, on management of sFGR in MC twin pregnancy will be assessed: power calculation, economic feasibility, identifying the research cohort (involving subtypes, gestation, intervention and control arms) and key outcomes.

A survey of clinicians will be performed as part of WP3 to identify current practices and opinion. Clinicians from the curated database of experts will be invited to participate in the survey. The results of this survey will be used to inform the selected consensus process approach that will ultimately formulate optimal trial design for use in a planned clinical trial in women with MC twin pregnancies complicated by sFGR. Depending on the results and findings of the previous WPs, we will take an adaptive approach when it comes to reaching a consensus on determining whether a future trial is possible (eg, this could take the form of a Delphi survey or a structured consensus meeting). Important issues and trial design constructs raised from these previous work streams will be formulated into a series of structured items for consideration. We will aim for at least 50% or more voting on the feasibility and acceptability of the study design/eligibility to take things forward as ‘plausible’. Plausibility will be defined as items where at least 50% of the voting participants deem the item as feasible and acceptable. An iterative process will be followed to allow further discussion on items when needed. The consensus opinion of relevant stakeholders on key preferred scenarios will be used to develop a protocol for this planned trial. The feasibility of this protocol will then be further consolidated using qualitative research and a final consensus process. If it becomes apparent that an RCT would not be feasible/acceptable, future research design would be agreed on by a structured consensus meeting.

### Statistical/data analysis considerations

Patients will be analysed on an intention-to-treat basis at the time of recruitment, retaining all patients irrespective of any protocol deviations. Further secondary analysis will be carried out on a per-protocol population. Further analyses may be carried out on planned subgroups (eg, those who meet the inclusion criteria for a future study) as required. As this is an exploratory study no formal levels of significance are set. All statistics will be presented alongside 95% CIs to give an indication of the level of precision. The likelihood of missing data is small given the standard procedure in place to manage the study centrally. Final analyses will take place on a complete-case basis with no adjustments made (eg, multiple imputation) in the case of missing data. Analysis of study data will take place once all participants have received the planned follow-up and all data are available for analysis. Continuous data will be summarised as median, IQR and ranges. Categorical data shall be summarised as frequencies of counts and associated percentages. Multivariate data analysis techniques will be used to attempt to find natural groupings in the generated data. In particular, hierarchical cluster analysis and principal component analysis techniques will be used.

Thematic analysis of qualitative data from the WP2 interviews and focus group(s) will be assisted by using NVivo V.12 qualitative data analysis package and SPSS, version 27 software for statistical analysis. While data will be analysed thematically, the focus will be modified to fit with the criterion of catalytic validity, whereby findings should be relevant to future research and practice (in particular, the design of the potential definitive RCT). Analysis will also draw on philosophical theory, concepts and methods (such as conceptual clarification and balanced argumentation) in an aim to develop recommendations that are defensible and consistent with key principles of biomedical ethics. This empirical ethics approach will facilitate the identification of practices or processes that should be challenged or modified in trial design. Quantitative analysis will involve simple descriptive statistics and the χ^2^ test for trends. Data from each method will be analysed separately then synthesised using constant comparative analysis. If it becomes apparent that an RCT would not be feasible/acceptable, future research design would be agreed on by a structured consensus meeting.

### Data monitoring

As there are no formal hypotheses being tested, there are no formal stopping rules (other than safety) or mechanisms defined here to stop the study prior to the planned end of the study. The study does have a formal independent oversight committee that will be able to review at regular intervals all accumulating data. The main responsibility of this committee will be to review the recruitment of participants, the collection of all essential data and to assess patient safety. The study oversight/steering committee will consist of (1) chief investigator, (2) independent clinician (chair), (3) research manager, (4) study statistician, (5) another independent clinician; (6) (patient and public involvement and engagement) coapplicant, (7) sponsor and (8) lead site representative. The role of the oversight/steering committee is to provide oversight of the study. In particular, this committee will concentrate on the progress of the study, adherence to the protocol, participant safety and consideration of new information.

### Ethics and dissemination

This study has received ethical approval from the Health Research Authority (HRA) Ethics Committee South West—Cornwall and Plymouth (REC reference 20/SW/0156, IRAS ID 286337), but all participating sites must undergo site-specific assessment of capacity and capability via the HRA. Any adverse events or protocol deviation will be identified and reported. The study will be conducted in accordance with, but not limited to, the Human Rights Act 1998, the Data Protection Act 2018, Freedom of Information Act 2000 subject to the provisions of sections 41 and 43 thereof, the EU (European Union) Clinical Trials Directive, ICH-GCP (International Council for Harmonisation Guideline for Good Clinical Practice), the Declaration of Helsinki 1996 and the UK Policy framework for Health and Social Care research as amended from time to time. All data will be recorded, collected, stored and processed, in accordance with GDPR(General Data Protection Regulation) (EU) 2016/679.

## References

[1] Draper ES, Gallimore ID, Kurinczuk JJ (2020). The Infant Mortality and Morbidity Studies, Department of Health Sciences.

[2] Scher AI, Petterson B, Blair E (2002). The risk of mortality or cerebral palsy in twins: a collaborative population-based study. Pediatr Res.

[3] Royal College of Obstetricians and Gynaecologists (2016). Management of monochorionic twin pregnancy. green-top guideline no.51.

[4] Khalil A, Beune I, Hecher K (2019). Consensus definition and essential reporting parameters of selective fetal growth restriction in twin pregnancy: a Delphi procedure. Ultrasound in Obstet & Gyne.

[5] Khalil A, Rodgers M, Baschat A (2016). ISUOG Practice guidelines: role of ultrasound in twin pregnancy. Ultrasound Obstet Gynecol.

[6] Gratacós E, Lewi L, Muñoz B (2007). A classification system for selective intrauterine growth restriction in monochorionic pregnancies according to umbilical artery Doppler flow in the smaller twin. Ultrasound Obstet Gynecol.

[7] Townsend R, D’Antonio F, Sileo FG (2019). Perinatal outcome of monochorionic twin pregnancy complicated by selective fetal growth restriction according to management: systematic review and meta‐analysis. Ultrasound in Obstet & Gyne.

[8] Buca D, Pagani G, Rizzo G (2017). Outcome of monochorionic twin pregnancy with selective intrauterine growth restriction according to umbilical artery Doppler flow pattern of smaller twin: systematic review and meta‐analysis. *Ultrasound in Obstet & Gyne*.

[9] Townsend R, Khalil A (2016). Twin pregnancy complicated by selective growth restriction. Curr Opin Obstet Gynecol.

[10] Monaghan C, Kalafat E, Binder J (2019). Prediction of adverse pregnancy outcome in monochorionic diamniotic twin pregnancy complicated by selective fetal growth restriction. Ultrasound Obstet Gynecol.

[11] Curado J, Sileo F, Bhide A (2020). Early- and late-onset selective fetal growth restriction in monochorionic diamniotic twin pregnancy: natural history and diagnostic criteria. Ultrasound Obstet Gynecol.

[12] Khalil A, Thilaganathan B (2019). Selective fetal growth restriction in monochorionic twin pregnancy: a dilemma for clinicians and a challenge for researchers. Ultrasound in Obstet & Gyne.

[13] Heazell AEP, Siassakos D, Blencowe H (2016). Stillbirths: economic and psychosocial consequences. Lancet.

[14] Ishii K, Nakata M, Wada S (2015). Feasibility and preliminary outcomes of fetoscopic laser photocoagulation for monochorionic twin gestation with selective intrauterine growth restriction accompanied by severe oligohydramnios. J Obstet Gynaecol Res.

[15] Chalouhi GE, Marangoni MA, Quibel T (2013). Active management of selective intrauterine growth restriction with abnormal Doppler in monochorionic diamniotic twin pregnancies diagnosed in the second trimester of pregnancy. Prenat Diagn.

[16] Inklaar MJ, van Klink JMM, Stolk TT (2014). Cerebral injury in monochorionic twins with selective intrauterine growth restriction: a systematic review. Prenat Diagn.

[17] Morsing E, Asard M, Ley D (2011). Cognitive function after intrauterine growth restriction and very preterm birth. Pediatrics.

[18] Marlow N, Wolke D, Bracewell MA (2005). Neurologic and developmental disability at six years of age after extremely preterm birth. N Engl J Med.

[19] Parra-Cordero M, Bennasar M, Martínez JM (2016). Cord occlusion in monochorionic twins with early selective intrauterine growth restriction and abnormal umbilical artery doppler: a consecutive series of 90 cases. Fetal Diagn Ther.

[20] Peng R, Xie H-N, Lin M-F (2016). Clinical outcomes after selective fetal reduction of complicated monochorionic twins with radiofrequency ablation and bipolar cord coagulation. Gynecol Obstet Invest.

[21] Malterud K, Siersma VD, Guassora AD (2016). Sample size in qualitative interview studies: guided by information power. Qual Health Res.

