# Supplementary material for: FERN: is it possible to conduct a randomised controlled trial of intervention or expectant management for early-onset selective fetal growth restriction in monochorionic twin pregnancy – protocol for a prospective multicentre mixed-methods feasibility study
Source: BMJ Open. 2024 Aug 17;14(8):e080021. doi: 10.1136/bmjopen-2023-080021 (PMC11331819; doi:10.1136/bmjopen-2023-080021)
Supplement: Supplementary file 1 [file bmjopen-2023-080021.reviewer_comments.pdf]

PEER REVIEW HISTORY

BMJ Open publishes all reviews undertaken for accepted manuscripts. Reviewers are asked to complete a checklist review form (**http://bmjopen.bmj.com/site/about/resources/checklist.pdf**) and are provided with free text boxes to elaborate on their assessment. These free text comments are reproduced below.

ARTICLE DETAILS

**TITLE (PROVISIONAL)**	FERN: Is it possible to conduct a Randomised controlled trial of Intervention or Expectant Management for Early Onset Selective Fetal Growth Restriction in Monochorionic Twin Pregnancy: protocol for a prospective multicentre mixed methods feasibility study
**AUTHORS**	Khalil, Asma; PRASAD, SMRITI; Woolfall, Kerry; Mitchell, Tracy; Kirkham, Jamie J.; Yaghi, Odai; Ricketts, Tracey; Attilakos, George; Bailie, Carolyn; Cornforth, Christine; Denbow, Mark; Hardman, Louise; Harrold, Jane; Parasuraman, Rajeswari; Leven, Shauna; Marsden, Joel; Mendoza, Jessica; Mousa, Tommy; Nanda, Surabhi; Thilaganathan, Baskaran; Turner, Mark; Watson, Michelle; Wilding, Karen; Popa, Mariana; Alfirevic, Zarko; Anumba, Dilly; Ashcroft, Richard; Baschet, Ahmet; da Silva Costa, Fabrício; Deprest, Jan; Fenwick, Natasha; Haak, Monique; Healey, Andy; Hecher, Kurt; Impey, Lawrence; Jackson, Richard; Johnstone, Edward; Lewi, Liesbeth; Lopriore, Enrico; Papageorghiou, Aris; Pasupathy, Dharmintra; Sandall, Jane; Sharp, Andrew; Thangaratinam, Shakila; Vollmer, B; Yinon, Yoav

VERSION 1 – REVIEW

**REVIEWER**	Segal, Nancy L.<br>Calif State Univ Fullerton
**REVIEW RETURNED**	06-Oct-2023

**GENERAL COMMENTS**	This paper lays out a plan for an important study in an important area, namely the best way of treating and managing discordance for Selective Fetal Growth Restriction in MZ twins. Three strategies, titled Work Packages, are described and labeled as WP1, WP2 and WP3.<br><br>I have only minor reservations which I am confident the authors can resolve.<br><br>--Please define FERN at the start; in fact, I do not recall seeing it defined anywhere.<br>--It is unclear if the purpose of this paper is to recruit participants or to describe the study.<br>It seems that it may be both.<br>--Under Methods and Analysis, it woold be clearer to readers if they bulleted WP1, 2, and 3, rather than having them in a paragraph; same for Strengths and Limitations.<br>--Assessing parents' preferences might include questions concerning parental<br>knowledge of sFGR. Some parents may have done some research in advance which could affect their decisions.

	--Women who decline to participate should be questions as to why.<br>--I realize the study focuses in MZ twins, btu perhaps brief mention might be made of DZ twins who are discordant for sFGR. Perhaps if their program works, it might be extended to these pairs and families.

**REVIEWER**	Gebb, Juliana<br>The Children's Hospital of Philadelphia
**REVIEW RETURNED**	16-Oct-2023

**GENERAL COMMENTS**	This is a well-written and interesting proposal. sFGR is an extremely important condition for which the best management is unknown. There are a lot of factors that play into the decisions that patients and providers make when treating the condition and I think this proposal is well-designed to capture the intricacies of the thought processes.

**REVIEWER**	Blumenfeld, Yair J.<br>Stanford University, Department of Obstetrics and Gynecology
**REVIEW RETURNED**	23-Oct-2023

**GENERAL COMMENTS**	• The investigators plan to study a specific segment of monochorionic twins, namely those with early onset sFGR (less than 24 weeks). Clearly a lot of time and effort is planned based on the protocol proposed. What is known about the incidence of this population (early onset sFGR in mono-di twins), and what is the disease burden, either clinically of financially? How many of the sFGR cases are currently diagnosed in the UK before 24 weeks? The incidence and outcomes described in the introduction section are mostly about all sFGR cases and not necessarily those before 24 weeks which is the focus of the study.<br><br>• I believe the authors plan to include all early onset sFGR cases, irrespective of Doppler abnormalities. This clinical manifestations and prognosis of this broad inclusion criteria could be quite varied. For example, there is a difference between a 19 week gestation sFGR (less than 2nd percentile) twin gestation with absent end diastolic flow vs. a 23 week gestation sFGR (9th percentile) with normal Doppler studies. How would the investigators account for this difference?<br><br>• The authors aim to assess the feasibility of conducting a future RCT. Much of the focus is on the clinical feasibility based on incidence and outcomes, but are there any ethical aspects to this feasibility? I’m assuming an element of the patient and clinician surveys in WP2 may address some ethical elements. If so, can the authors expand on this dimension a bit.<br><br>• The authors state that one of the exclusions in WP1 will be a known karyotype abnormality. Will an amniocentesis be mandated for enrollment? If not, how will the authors account for genetic anomalies (or event structural anomalies) discovered after 24 weeks or even after birth?<br><br>• For WP1, how is “active fetal intervention” defined? RFA, bipolar coagulation and laser? Or without laser?

	• Do all participating centers offer active management as an option currently? If not, how many of the centers do? Could there be knowledge gaps between centers and providers at the centers based on whether they currently provide active management?<br><br>• For WP2 please define "clinicians". Are these physicians or other allied health professionals? If the latter how will the investigators combine input from different provider types and different patients who may or may not be equally knowledgeable about the clinical management dilemma?

**REVIEWER**	Oluyomi, Titilayo<br>University of Calgary Cumming School of Medicine
**REVIEW RETURNED**	31-Oct-2023

**GENERAL COMMENTS**	This is an excellent idea.<br>My comments are as follows:<br>1) There does not appear to be an option of not doing a study at all if the surveys in WKp 1 and 2 deem this study not feasible. There only appears to be a plan to change or adjust the study design. There also needs to be an option to say the study is not feasible in any form (though this outcome is unlikely).<br>2) Also using 50% as the threshold for acceptability of the study by survey participants, is just like rolling dice - we then almost don't need the study. This is a VERY delicate matter, I would suggest an acceptability level of at least 70 % for most aspects of the study EXCEPT for things like type 3 sIUGR which really has no clear management or outcome (in which case 50% is acceptability rate is fair)<br>3)I was wondering if the randomization groups could be amended as follows;<br>Expectant management only for type 3 sIUGR (when no deterioration). This might also be addressed in your consensus development phase when you talk about subgroups.<br>Voting % in the 3rd phase needs to be higher (~70%) to consider this plausible/feasible. I agree with this statement: "If it becomes apparent that an RCT would not be feasible/acceptable, future research design would be agreed upon by a structured consensus meeting."

**REVIEWER**	Ambroise Grandjean, Gaëlle<br>Université de Lorraine, INSERM
**REVIEW RETURNED**	18-Dec-2023

**GENERAL COMMENTS**	Title	1	The title allows identifying the study design, population, and interventions.<br><br>Add the mention "protocol" to limit confusion with the further publications
	Funding	23	Sources of financial
	**Introduction**		

	Background and rationale	6a	The research question and justification for the RCT are relevant (examining the benefits and harms of each intervention). The need for a feasibility study is clearly stated/
	Objectives	7	Specific objectives for each Work package are clearly stated
	Trial design	8	Description of future trial design requires precisions on intervention group (termination ? selective termination ? Placental laser photocoagulation ?)
	**Methods: Participants, interventions, and outcomes**		
	Study setting	12	The description of study settings needs to be completed regarding the centre's characteristics (community clinic, academic hospital). Maybe provide a reference to where the list of study sites can be obtained
	Interventions	13 - 14	Please, be more specific on data collection for WP1 (which outcomes are intended? How long will the follow-up continue after demise or childbirth?<br><br>Please be more specific for WP2; who will drive the interview and focus group?
		13	To better understand acceptability, specify which maternal characteristics are to be collected (socio-economic background)
	Outcomes	12	For WP1, specify prespecified criteria to judge whether or how to proceed with a future definitive trial (rate of missing data ?)
	Data management	19	For WP1, specify the data entry, coding, security, storage, and monitoring plans. Would the data be collected prospectively or retrospectively? Who will collect the data?
	**Methods: Monitoring**		

	Data monitoring 21a Specify the composition of the data monitoring committee. Alternatively, an explanation of why a DMC is not needed<br><br>**Ethics and dissemination**<br><br>Research ethics approval 24 Plans for seeking research ethics committee/institutional review board (REC/IRB) approval<br><br>Declaration of interests 28 Financial and other competing interests for principal investigators for the overall trial and each study site<br><br>**Appendices**<br><br>Informed consent materials n/a Provide the model consent form and other related documentation given to participants<br><br>Biological specimens n/a For WP2, provide the interview grid for patients' and clinicians' views exploration<br><br>**Discussion** n/a Specify the pilot trial limitations, addressing sources of potential bias and remaining uncertainty about feasibility in a specific paragraph<br><br>External validity and applicability to the future settings for RCT<br><br>The manuscript presents the protocol of a feasibility study.<br><br>Background and rationale are legitimated, and the objectives are clearly stated.

	The expected impact is limited to a small population (monochorionic with SFGR), yet with a potentially major benefit for patients and families.<br><br>The protocol is clear but requires some additional information to be assessed entirely and to guarantee intervention reproducibility. The following points result from a first analysis through SPIRIT and CONSORT checklists and should be addressed in priority to optimise the external validity of the protocol and identify and discuss potential bias.

VERSION 1 – AUTHOR RESPONSE

Reviewer: 1

Dr. Nancy L. Segal, Calif State Univ Fullerton

Comments to the Author:

This paper lays out a plan for an important study in an important area, namely the best way of treating and managing discordance for Selective Fetal Growth Restriction in MZ twins. Three strategies, titled Work Packages, are described and labeled as WP1, WP2 and WP3.

I have only minor reservations which I am confident the authors can resolve.

--Please define FERN at the start; in fact, I do not recall seeing it defined anywhere.

Response: We have defined FERN at the first instance (title of the manuscript) now which is a restructured acronym for the title of the study - Intervention or Expectant Management for Early Onset Selective Fetal Growth Restriction in Monochorionic Twin Pregnancy

--It is unclear if the purpose of this paper is to recruit participants or to describe the study.

It seems that it may be both.

Response: The purpose of this paper is to describe the study protocol. To clarify this, we have amended the title of the manuscript.

--Under Methods and Analysis, it woold be clearer to readers if they bulleted WP1, 2, and 3, rather than having them in a paragraph, same for Strengths and Limitations.

Response: We have changed the format to bulleted points as requested by the reviewer. Strengths and limitations are in a bulleted format in the original manuscript.

--Assessing parents' preferences might include questions concerning parental knowledge of sFGR. Some parents may have done some research in advance which could affect their decisions.

Response: We thank the reviewer for their comment. Please see the questions in the attached 'Mother and Birth Partner Interview Topic Guide'. The questions were more about the two interventions (laser/cord occlusion) than about sFGR.

--Women who decline to participate should be questions as to why.

Response: The WP2 interviews will include these questions. Mothers and birth partners will be asked: 'Would you have given your permission to take part in the FERN study?... Could you tell me a bit more about your reasons for this?' and then asked questions about each proposed arm: How would you feel if you were randomised to 'watch and wait' (expectant management) in the proposed FERN trial? How would you feel if you were randomised to receive cord occlusion (selective termination) in the proposed FERN trial? How would you feel if you were randomised to receive laser treatment in the proposed FERN trial?" This is explained in the Interview guide attached as supplementary material.

--I realize the study focuses in MZ twins, btu perhaps brief mention might be made of DZ twins who are discordant for sFGR. Perhaps if their program works, it might be extended to these pairs and families.

Response: We respectfully disagree with the reviewer. Management options and dilemmas in sFGR for monochorionic twin pregnancies are different from dizygotic twin pregnancies, therefore the results from this study will not be applicable to DZ twins.

Reviewer: 2

Dr. Juliana Gebb, The Children's Hospital of Philadelphia

Comments to the Author:

This is a well-written and interesting proposal. sFGR is an extremely important condition for which the best management is unknown. There are a lot of factors that play into the decisions that patients and providers make when treating the condition and I think this proposal is well-designed to capture the intricacies of the thought processes.

Response: We thank the reviewer for their positive comment.

Reviewer: 3

Dr. Yair J. Blumenfeld, Stanford University

Comments to the Author:

• The investigators plan to study a specific segment of monochorionic twins, namely those with early onset sFGR (less than 24 weeks). Clearly a lot of time and effort is planned based on the protocol proposed. What is known about the incidence of this population (early onset sFGR in mono-di twins), and what is the disease burden, either clinically of financially? How many of the sFGR cases are currently diagnosed in the UK before 24 weeks? The incidence and outcomes described in the introduction section are mostly about all sFGR cases and not necessarily those before 24 weeks which is the focus of the study.

Response: We thank the reviewer for their comment. We have published on the natural history of sFGR in another paper (Curado J, Sileo F, Bhide A, Thilaganathan B, Khalil A. Early- and late-onset selective fetal growth restriction in monochorionic diamniotic twin pregnancy: natural history and diagnostic criteria. Ultrasound Obstet Gynecol. 2020 May;55(5):661–6.). The incidence and natural history of sFGR is not the subject of this study, therefore, not elaborated in detail, however as suggested by the reviewer, we have now included this reference in the paper.

• I believe the authors plan to include all early onset sFGR cases, irrespective of Doppler abnormalities. These clinical manifestations and prognosis of this broad inclusion criteria could be quite varied. For example, there is a difference between a 19 week gestation sFGR (less than 2nd percentile) twin gestation with absent end diastolic flow vs. a 23 week gestation sFGR (9th percentile) with normal Doppler studies. How would the investigators account for this difference?

Response: We thank the reviewer for this comment. We agree that the prognosis of sFGR can be quite varied, and sometimes it is unclear whether changes in Doppler flow patterns are a manifestation of progression of the condition or mere variation. These cases have been subclassified based on the Umbilical artery Doppler flow patterns into Type I, II and III. We will perform a sub-group analysis of the data collected according to the gestational age at onset and Umbilical artery Doppler flow patterns.

• The authors aim to assess the feasibility of conducting a future RCT. Much of the focus is on the clinical feasibility based on incidence and outcomes, but are there any ethical aspects to this feasibility? I'm assuming an element of the patient and clinician surveys in WP2 may address some ethical elements. If so, can the authors expand on this dimension a bit.

Response: We thank the reviewer for this comment. As the reviewer has pointed out, the WP2 will address and identify the ethical dilemmas' clinicians and parents face while making decisions.

Additionally, we have submitted an opinion article that is under consideration for publication and outlines the ethical dimensions, pertinent to this condition.

• The authors state that one of the exclusions in WP1 will be a known karyotype abnormality. Will an amniocentesis be mandated for enrollment? If not, how will the authors account for genetic anomalies (or event structural anomalies) discovered after 24 weeks or even after birth?

Response: Amniocentesis (Testing for genetic abnormality) is not mandated for enrolment as this is not part of the routine care of these pregnancies – selective fetal growth restriction in monochorionic pregnancies is largely attributed to unequal sharing of a single placenta. If genetic abnormalities are discovered after 24 weeks/birth or discovered before 24 weeks as the test was performed for clinical reason/parental choice, that case will be excluded from the study.

• For WP1, how is "active fetal intervention" defined? RFA, bipolar coagulation and laser? Or without laser?

Response: For WP1, ''active intervention" is defined as i) Laser photocoagulation, or ii) Selective termination (which can be performed by either RFA or bipolar cord coagulation). We have added a line in the text to clarify it.

• Do all participating centers offer active management as an option currently? If not, how many of the centers do? Could there be knowledge gaps between centers and providers at the centers based on whether they currently provide active management?

Response: All the centres provide active management – either primarily, at the centre itself, or at the tertiary referral units where these women are referred for active intervention. The list of proposed participating centres is provided in the supplementary material.

• For WP2 please define "clinicians". Are these physicians or other allied health professionals? If the latter how will the investigators combine input from different provider types and different patients who may or may not be equally knowledgeable about the clinical management dilemma?

Response: "Clinicians" are physicians involved in the care of these pregnancies.

Reviewer: 4

Dr. Titilayo Oluyomi, University of Calgary Cumming School of Medicine

Comments to the Author:

This is an excellent idea.

My comments are as follows:

1.There does not appear to be an option of not doing a study at all if the surveys in WKp 1 and 2 deem this study not feasible. There only appears to be a plan to change or adjust the study design. There also needs to be an option to say the study is not feasible in any form (though this outcome is unlikely).

Response: We respectfully disagree with the comment. The protocol specifies that in the event, the study (in the context of FERN project, this would be an RCT) is deemed not feasible, we will explore alternative study designs, other than an RCT.

2. Also using 50% as the threshold for acceptability of the study by survey participants, is just like rolling dice - we then almost don't need the study. This is a VERY delicate matter, I would suggest an acceptability level of at least 70 % for most aspects of the study EXCEPT for things like type 3 sIUGR which really has no clear management or outcome (in which case 50% is acceptability rate is fair)

Response: We thank the reviewer for this comment. We wanted to keep the threshold particularly low to not rule out the possibility of conducting a trial in this field too hastily. If more than half felt that it was acceptable, then perhaps this warranted further discussion and further voting. Also, as the reviewer points out – for conditions, for example, Type III sFGR which has no clear management or outcome – 50% acceptability rate is fair.

3. I was wondering if the randomization groups could be amended as follows;

Expectant management only for type 3 sIUGR (when no deterioration). This might also be addressed in your consensus development phase when you talk about subgroups.

Voting % in the 3rd phase needs to be higher (~70%) to consider this plausible/feasible. I agree with this statement: "If it becomes apparent that an RCT would not be feasible/acceptable, future research design would be agreed upon by a structured consensus meeting."

Response: We thank the reviewer for their suggestion, but we would like to not pre-empt the results, therefore, this has not been included in the protocol.

Reviewer: 5

Mrs. Gaëlle Ambroise Grandjean, Université de Lorraine, CHU Nancy

Comments to the Author:

The manuscript presents the protocol of a feasibility study.

Background and rationale are legitimated, and the objectives are clearly stated.

The expected impact is limited to a small population (monochorionic with SFGR), yet with a potentially major benefit for patients and families.

The protocol is clear but requires some additional information to be assessed entirely and to guarantee intervention reproducibility. The following points result from a first analysis through SPIRIT and CONSORT checklists and should be addressed in priority to optimise the external validity of the protocol and identify and discuss potential bias.

Response: We have provided the responses alongside the queries raised (see below)

			**Response to the comments**
Title	1	The title allows identifying the study design, population, and interventions.<br><br>Add the mention "protocol" to limit confusion with the further publications	*We have amended the title of the manuscript to include "protocol".*
Funding	23	Sources of financial	*Funding statement is provided in the manuscript –"This work is funded by National Institute for Health and Care Research (NIHR) via grant number NIHR128596.*
**Introduction**			
Background and rationale	6a	The research question and justification for the RCT are relevant (examining the benefits and harms of each intervention). The need for a feasibility study is clearly stated/	*NA*
Objectives	7	Specific objectives for each Work package are clearly stated	*NA*
Trial design	8	Description of future trial design requires precisions on intervention group (termination ? selective termination ? Placental laser photocoagulation ?)	*Active intervention is defined as:*<br><br>*1) Laser photocoagulation*<br><br>*2)Selective termination (which can be performed by either RFA or*<br><br>*bipolar cord coagulation).*

**Methods: Participants, interventions, and outcomes**			
Study setting	12	The description of study settings needs to be completed regarding the centre's characteristics (community clinic, academic hospital). Maybe provide a reference to where the list of study sites can be obtained	*We have provided a list of proposed centres in the supplementary Table S1 with their characteristics.*
Interventions	13 - 14	Please, be more specific on data collection for WP1 (which outcomes are intended? How long will the follow-up continue after demise or childbirth?<br><br>Please be more specific for WP2; who will drive the interview and focus group?	*The data will be collected on a range of data outcomes which is described on the FERN REDCap database. We have also attached the final FERN Case Report Form as a supplementary material. The follow-up will continue till the date of Discharge of baby/babies from the hospital/neonatal unit.*<br><br>*Above has been added to the manuscript*<br><br>*Research Associates from the University of Liverpool (Dr Tracy Mitchell; Mariana Popa), led by Dr Kerry Woolfall will drive the interview and focus group.*
	13	To better understand acceptability, specify which maternal characteristics are to be collected (socio-economic background)	*The Case Report Form is attached as a supplementary file, which specifies the maternal characteristics which would be collected.*
Outcomes	12	For WP1, specify prespecified criteria to judge whether or how to proceed with a future definitive trial (rate of missing data?)	*Whether or not to proceed with a future definitive trial will depend not only on WP1, but all information from WP1 and WP2 will be fed into WP3 consensus Meeting /Delphi.*

Data management	19	For WP1, specify the data entry, coding, security, storage, and monitoring plans. Would the data be collected prospectively or retrospectively? Who will collect the data?	*The data will be collected on a range of data outcomes which is described on the FERN REDCap database. The follow-up will continue till the date of discharge of baby/babies from the hospital/neonatal unit. This database is designed and maintained by the University of Liverpool, IT Services in collaboration with the Chief Investigator and Research Manager. The eCRF (electronic Case Record Form) is the primary data collection instrument for the study. All data requested on the eCRF must be recorded and all missing data explained. WP1 is a prospective observational study therefore, all data will be collected prospectively by trained members of the Research team at each study site.*
**Methods: Monitoring**			
Data monitoring	21a	Specify the composition of the data monitoring committee. Alternatively, an explanation of why a DMC is not needed	*As no formal hypotheses are being tested, there are no formal stopping rules other than safety) or mechanisms defined here to stop the study prior to the planned end of study. The study does have a formal independent oversight committee that will be able to review at regular intervals all accumulating data.*<br><br>*The main responsibility of this committee will be to review the recruitment of participants, the collection of all essential data and to assess patient safety.*<br><br>*The Study Oversight / Steering Committee will consist of:*<br>*CI / PI;*<br>*Independent Clinician (Chair);*<br>*Research Manager;*<br>*Study Statistician;*<br>*One further Independent Clinician;*<br>*PPIE Co Applicant;*<br>*Sponsor; and*<br>*Lead Site Representative*<br><br>*The role of the Oversight / Steering Committee is to provide oversight of the study. In particular, this committee will concentrate on the progress of the study, adherence to the protocol, participant safety and consideration of new information.*<br>*We have added a segment on data monitoring to the manuscript.*
**Ethics and dissemination**			

Research ethics approval	24	Plans for seeking research ethics committee/institutional review board (REC/IRB) approval	*This study has received ethical approval from the Health Research Authority (HRA) South West - Cornwall and Plymouth Ethics Committee (REC reference 20/SW/0156, IRAS ID 286337).*<br><br>*All participating sites will undergo site-specific approvals for assessment of capacity and capability by the HRA. This is mentioned in the original manuscript.*
Declaration of interests	28	Financial and other competing interests for principal investigators for the overall trial and each study site	*There are no financial or other competing interests for principal investigators.*<br><br>*This is mentioned in the manuscript*
**Appendices**			
Informed consent materials	n/a	Provide the model consent form and other related documentation given to participants	*We have provided the participant information sheet and consent form as Supplementary files*
Biological specimens	n/a	For WP2, provide the interview grid for patients' and clinicians' views exploration	*This has been provided as a supplementary file.*
**Discussion**	n/a	Specify the pilot trial limitations, addressing sources of potential bias and remaining uncertainty about feasibility in a specific paragraph	*The limitations have been added to the Strengths and Limitations sections.*
		External validity and applicability to the future settings for RCT	
